# Effect of Salivary Exosomal miR-25-3p on Periodontitis With Insulin Resistance

**DOI:** 10.3389/fimmu.2021.775046

**Published:** 2022-01-07

**Authors:** Jin-Seok Byun, Ho Yeop Lee, Jingwen Tian, Ji Sun Moon, Jaejin Choi, Sang-Hee Lee, Yong-Gun Kim, Hyon-Seung Yi

**Affiliations:** ^1^ Department of Oral Medicine, Kyungpook National University School of Dentistry, Daegu, South Korea; ^2^ Department of Medical Science, Chungnam National University School of Medicine, Daejeon, South Korea; ^3^ Research Center for Endocrine and Metabolic Diseases, Chungnam National University School of Medicine, Daejeon, South Korea; ^4^ Laboratory of Endocrinology and Immune System, Chungnam National University School of Medicine, Daejeon, South Korea; ^5^ Department of Research and Development, Panagene Inc., Daejeon, South Korea; ^6^ Bio-Electron Microscopy Research Center (104-Dong), Korea Basic Science Institute, Cheongju, South Korea; ^7^ Department of Periodontology, Kyungpook National University School of Dentistry, Daegu, South Korea

**Keywords:** periodontitis, miRNA, exosome, saliva, diabetes, insulin resistance, inflammation

## Abstract

Periodontitis is caused by an oral microbial dysbiosis-mediated imbalance of the local immune microenvironment, which is promoted by insulin resistance and obesity. The prevalence and severity of periodontitis is higher in patients with type 2 diabetes than in healthy individuals, possibly because of differences in immune responses. The level of glycemic control also affects the saliva profile, which may further promote periodontal disease in diabetes patients. Therefore, we compared the salivary exosomal miRNA profiles of patients with type 2 diabetes with those of healthy individuals, and we found that exosomal miR-25-3p in saliva is significantly enriched (by approximately 2-fold, *p* < 0.01) in obese patients with type 2 diabetes. We also identified CD69 mRNA as a miR-25-3p target that regulates both activation of γδ T cells and the inflammatory response. Knockdown of CD69 increased (by approximately 2-fold) interleukin-17A production of γδ T cells *in vitro*. To evaluate the role of exosomal miRNA on progression of periodontitis, we analyzed regional immune cells in both periodontal tissues and lymph nodes from mice with periodontitis. We found that diet-induced obesity increased the population of infiltrating pro-inflammatory immune cells in the gingiva and regional lymph nodes of these mice. Treatment with miR-25-3p inhibitors prevented the local *in vivo* inflammatory response in mice with periodontitis and diet-induced obesity. Finally, we showed that suppression of interleukin 17-mediated local inflammation by a miR-25-3p inhibitor alleviated (by approximately 34%) ligature-induced periodontal alveolar bone loss in mice. Taken together, these data suggest that exosomal miR-25-3p in saliva contributes to development and progression of diabetes-associated periodontitis. Discovery of additional miR-25-3p targets may provide critical insights into developing drugs to treat periodontitis by regulating γδ T cell-mediated local inflammation.

## Introduction

Periodontitis is a common chronic inflammatory disease that is primarily caused by the host inflammatory response to the bacterial challenge presented by the biofilm. The pathogenic mechanism of periodontitis is an oral microbial dysbiosis-mediated imbalance of the local immune microenvironment, which may develop intermittently into a systemic inflammatory response (1). Inflammation is a key pathogenic feature of both periodontitis and diabetes, and diabetes patients who poorly control their glycemic response are at greater risk for periodontitis than normoglycemic individuals (2, 3). Conversely, periodontal inflammation can increase the level of glycosylated hemoglobin and (subsequently) cause either prediabetes or overt diabetes (4). Active periodontal therapy improves glycemic control in patients with type 2 diabetes (T2D) (5). However, the precise mechanisms that underpin the links between diabetes and periodontitis are not completely understood.

Saliva has been used to assess development and severity of periodontal disease. Saliva includes many kinds of proteins and peptides that protect against periodontopathogenic infection (6). The composition of the salivary microbiome is also associated with periodontal health (7). Diverse markers from whole saliva are used to predict soft-tissue inflammation and alveolar bone loss (6). Recently, salivary exosomes (which contain proteins, lipids, mRNAs, and microRNAs) have emerged as potential biomarkers of systemic diseases (such as cancer and metabolic disease) (8). By facilitating both local and distal intercellular communication, exosomes may be important for development and progression of many diseases. However, it is unclear whether salivary exosomes play a role in diabetes-associated periodontitis.

Interleukin (IL)-17 producing T cells contribute to the pathogenesis of periodontal inflammation and bone loss (9). Although IL-17 defends against microbial infections by further inducing pro-inflammatory cytokines, it also increases insulin resistance and apoptosis of beta cells, which hamper glycemic control in T2D patients (10). T_H_17 cells and IL-17-producing γδ T cells are abundant in periodontal lesions, and the abundance of these types of cells in lesions correlate with the severity of periodontitis (11). Inflammation (mediated by IL-17-producing T cells) is important for development of periodontitis, but a salivary exosome-based regulatory mechanism has not been identified.

In this study, we assessed the composition of salivary exosomal miRNAs in both T2D patients and in healthy individuals. We demonstrate that insulin resistance alters the profile of exosomal miRNAs in saliva, which contributes to the progression of periodontitis in mice by modulating IL-17-mediated inflammation in both periodontal and regional lymph nodes. These findings suggest that exosomal miRNAs are key factors for the development and progression of diabetes-associated periodontitis in mice.

## Materials and Methods

### Study Participants

Thirty patients with type 2 diabetes (T2D) and 30 healthy individuals were recruited in Chungnam National University Hospital (CNUH) between October 2018 and April 2019. Patients with any of the following conditions were excluded from the study: severe pulmonary disease; acute or chronic kidney disease (estimated glomerular filtration rate < 30 mL/min/1.73 m^2^); any malignant or autoimmune disease; and liver disease with high levels of plasma aspartate transaminase or alanine transaminase (> 80 IU/L); smoking; active periodontitis and oral inflammatory diseases; or a history of radiotherapy for head and neck cancer. All participants were asked to refrain from eating and drinking for an hour prior to donating saliva samples. The participants were asked to sit in a comfortable position and rinse their mouths with bottled water to remove food debris. Saliva was collected from each participant in a 50-mL sterile Falcon tube (Becton, Dickinson and Company, New Jersey, USA). Immediately after collection, the saliva was placed on ice and transferred to a deep freezer. The study was reviewed and approved by the Institutional Review Board of CNUH (CNUH 2015-09-042), according to the standards of the Declaration of Helsinki. Written and oral informed consent, documented by the Department of Internal Medicine of CNUH in South Korea, was obtained from all of the participants prior to their inclusion in the study.

### Mice

Wild-type C57BL/6-background mice were purchased from the Jackson Laboratory (Bar Harbor, ME, USA) and maintained in a specific pathogen-free animal facility (CNUH Preclinical Research Center) in a controlled environment (light cycle, 12 h light/12 h dark; humidity, 50–60%; ambient temperature, 22 ± 2°C). All animal experiments were approved by the Institutional Review Board on Animal Experimentation of Chungnam National University School of Medicine (CNUH-019-A0071) and performed in accordance with the guidelines and regulations of Chungnam National University.

### *Ligature-*Induced Periodontitis Model *and Synchrotron* Radiation Micro-Computed Tomography *(SR-µCT)*

Ten male C57BL/6 mice were housed in an animal breeding room for 12 weeks. Five mice were fed a high-fat diet (HFD), and the other five mice were fed a normal chow diet (NCD). After 12 weeks, all mice were anesthetized for ligation with 7-0 non-absorbable braid silk (Ailee, Busan, South Korea). On day 1, silk was ligated around the second molar of the left-side maxilla of each mouse; but not around that of the right side. On day 9, the mice were sacrificed, and whole maxilla tissues (including left and right teeth) were collected and fixed in 4% paraformaldehyde. In addition, regional lymph nodes and gingival tissues were prepared for fluorescence-activated cell sorting (FACS) analysis of infiltrating immune cells. For experiments involving insulin resistance-associated periodontitis, male mice fed a HFD for 12 weeks were treated every other day with either saline (n = 5) or miR-25-3p inhibitors (n = 5; 100 μl of 8 nM), starting 3 days after the molar ligation procedure. SR-µCT was used to analyze changes in alveolar bone height. SR-µCT scans were performed at the beamline of the Biomedical Imaging of the Pohang Light Source II by using 23 keV of x-ray energy 400 mA of beam current. The field of view was 1.6 mm (horizontal) × 1.4 mm (vertical), and the effective pixel size was 0.65 μm. Alveolar bone height was defined as the distance between the cemento-enamel junction and the alveolar bone crest. To assess alveolar bone loss in the molar area, 3D coronal section images obtained by micro-CT data were used, and the bone area was measured by using Amira software, version 6.2 (FEI Co., Hillsboro, OR, USA).

### *FACS* Analysis

Murine periodontal tissues were prepared as described previously (12). Isolated mononuclear cells from submandibular lymph nodes and periodontal lesions of either ligated or non-ligated regions were resuspended in Dulbecco’s phosphate-buffered saline (Welgene, Daegu, South Korea) containing 0.5% BSA and 0.05% sodium azide, and then labeled with FACS antibodies. To block non-specific binding, cells were pre-incubated with mouse CD16/32 Fc block (eBioscience/ThermoFisher Scientific, Waltham, MA, USA) before labeling with FACS antibodies. Cells were also stained with eFluor 780-labeled Fixable Viability Dye (eBioscience/ThermoFisher Scientific) to exclude dead cells from FACS analysis. Lymphocytes (CD4 T, CD8 T, and regulatory T cells), monocytes, and neutrophils were analyzed by using anti-CD45, anti-CD4, anti-CD8, anti-CD11b, anti-CD44, anti-CD62L, anti-CD279, anti-TCRγδ, anti-CD25, and anti-Foxp3 antibodies (eBioscience/ThermoFisher Scientific). The Foxp3 Staining Buffer Set (eBioscience/ThermoFisher Scientific) was used to stain intracellular Foxp3. For intracellular cytokine staining, cells were stimulated with phorbol-myristate acetate/ionomycin/brefeldin A/monensin for 5 hours *in vitro* and then fixed and permeabilized by using a Fixation/Permeabilization Buffer kit (eBioscience/ThermoFisher Scientific). Permeabilized cells were washed with FACS buffer and resuspended in 1% formaldehyde and stained for intracellular cytokines with fluorochrome‐conjugated anti-TNF‐α and anti-IL‐17A antibodies. Stained cells were analyzed by using a BD LSRFortessa flow cytometer (BD Biosciences, San Jose, CA, USA), and data were analyzed using FlowJo software, v10 (FlowJo, LLC, Ashland, OR, USA).

## Results

### High-Fat Diet Induces an Inflammatory Response in the Regional Lymph Nodes of Mice With Periodontitis

**Figure 1:**
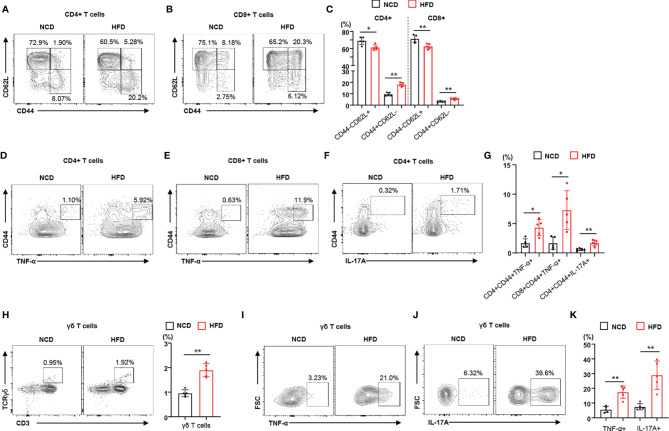
Diet-induced obesity promotes the local inflammatory response in male mice with periodontitis. **(A–C)** Representative flow cytometry plots and percentages of CD44^+^CD62L^−^ and CD44^−^CD62L^−^ cells in the CD4^+^ and CD8^+^ T cell populations from submandibular lymph nodes of either NCD-fed (n = 5) or HFD-fed (n = 5) male mice with periodontitis 9 days after ligature placement. **(D–G)** Frequency of TNF-α- or IL-17A-producing cells in the populations of CD4^+^ or CD8^+^ T cells in periodontitis-induced mice fed with either a NCD or a HFD. **(H)** Frequency of γδ T cells in submandibular lymph nodes of periodontitis-induced mice fed with either a NCD or a HFD. **(I–K)** Representative flow cytometry plots and percentages of either TNF-α- or IL-17A-producing cells in the population of γδ T cells from periodontitis-induced mice fed with either a NCD or a HFD. Data represent mean values of more than three independent experiments. Data are expressed as the mean ± SEM. *p < 0.05, **p < 0.01 [**(C, G, H, K)**: two‐tailed *t* tests].

To identify the immune phenotype of obesity-induced insulin resistance leading to local inflammation, we used a periodontitis mouse model that recapitulated the effects of diet-induced obesity and insulin resistance on the severity of disease. We investigated the immunophenotype of lymphocytes and γδ T cells in regional lymph nodes of mice with ligature-induced periodontitis (**Supplementary Figure 1**). We observed that HFD-fed mice gained more weight than NCD-fed mice (**Supplemental Figure 2A**). Analyzing a subset of CD4^+^ and CD8^+^ T lymphocytes, we observed that the population of memory T cells (CD44^+^CD62L^–^) increased in the regional lymph nodes of HFD-fed mice with periodontitis, whereas the population of naïve T cells (CD44^–^CD62L^+^) decreased (**Figures 1A–C**). We also found that the populations of naïve and memory T cells in NCD-fed mouse control (non-ligated) lymph nodes were not significantly different from those in HFD-fed mouse control lymph nodes (**Supplemental Figure 2B**). To address the contribution of diet-induced obesity to the functional characteristics of lymphocytes, we measured the populations of pro-inflammatory cytokine-producing CD4^+^ and CD8^+^ T cells in the regional lymph nodes of mice fed either a NCD or a HFD. The populations of TNF-α- or IL-17A-producing memory CD4^+^ and CD8^+^ T cells were significantly higher in the regional lymph nodes of HFD-fed mice with periodontitis than in those of mice fed a NCD (**Figures 1D–G**). We found significantly more TNF-α- or IL-17A-producing CD4^+^ and CD8^+^ T cells in the regional lymph nodes of mice with ligature-induced periodontitis than in control lymph nodes (**Supplementary Figure 2C**). In addition, γδ T cells were more highly enriched in the regional lymph nodes of mice fed a HFD (**Figure 1H**). The proportion of TNF-α- or IL-17-producing γδ T cells were significantly elevated in the regional lymph nodes of a HFD-fed mice with periodontitis (**Figures 1I–K**). Taken together, these results indicate that diet-induced obesity and insulin resistance increases the populations of infiltrating pro-inflammatory immune cells in the regional lymph nodes of mice with periodontitis.

### MiRNA Profiles of Salivary Exosomes in Healthy Individuals and in T2D Patients

Diabetes is a major risk factor for periodontal inflammation, whose development is facilitated by local and systemic pro-inflammatory cytokines (13). Saliva composition is associated with both oral disease (e.g. periodontal disease) and systemic disease (e.g. diabetes and insulin resistance) and can also affect disease progression (14). Diabetes compromises salivary gland function, which changes the biochemical characteristics of saliva and the salivary proteome and transcriptome (15). Since obese mice with insulin resistance had aggravated local inflammation and periodontitis-induced bone loss (**Supplementary Figure 1**), we recruited 60 participants (30 healthy individuals and 30 T2D patients) to isolate and analyze exosomes from whole saliva. The mean age of healthy individuals was 55.1 ± 10.4 years, and that of T2D patients was 56.3 ± 14.9 years (P = 0.352; **Table 1**). We measured baseline characteristics of the healthy individuals (including 13 men, 43.3%) and T2D patients (including 14 men, 46.7%) (**Table 1**). T2D patients had higher body mass indices and higher serum levels of HbA1c, fasting blood sugar, and 2-hour postprandial glucose than healthy individuals (**Table 1** and **Supplementary Figures 3A–D**). We treated all T2D patients only with antidiabetic drugs. The prevalence of smoking, a major risk factor of periodontal disease, was similar between the two groups, but the T2D patients were more susceptible to periodontal disease than were healthy individuals (**Table 1**).

**Table 1:** Clinical characteristics and serum chemistry of the study population.

Variables	Healthy individuals	Patients with Type 2 diabetes	P-value
Age, y	55.1 ± 10.4	56.3 ± 14.9	0.352
Male, n (%)	13, (43.3%)	14, (46.7%)	0.400
BMI, kg/m^2^	23.4 ± 2.32	26.5 ± 2.99	<0.001
FBS, mg/dL	91.2 ± 7.43	154.5 ± 35.0	<0.001
PP2, mg/dL	143.7 ± 22.3	230.3 ± 89.4	<0.001
HbA1c, %	5.61 ± 0.65	9.49 ± 1.80	<0.001
AST, IU/L	22.0 ± 7.12	24.3 ± 13.9	0.211
ALT, IU/L	26.1 ± 8.38	29.6 ± 25.3	0.235
Total cholesterol, mg/dL	155.2 ± 39.1	175.5 ± 41.5	0.028
Triglyceride, mg/dL	146.7 ± 39.4	235.4 ± 161.7	0.003
HDL cholesterol, mg/dL	50.7 ± 12.3	44.7 ± 11.5	0.029
LDL cholesterol, mg/dL	91.3 ± 23.4	104.8 ± 31.6	0.032
Creatinine, mg/dL	0.78 ± 0.20	0.92 ± 0.53	0.093
Hx of periodontal disease, n (%)	5, (16.7%)	13, (43.3%)	0.012
Smoking, n (%)	9, (30%)	8, (26.7%)	0.390

BMI, body mass index; FBS, fasting blood sugar; PP2, 2-hour postprandial glucose; AST, aspartate transaminase; ALT, alanine transaminase; HDL, high-density lipoprotein; LDL, low-density lipoprotein; Hx, history.

**Figure 2:**
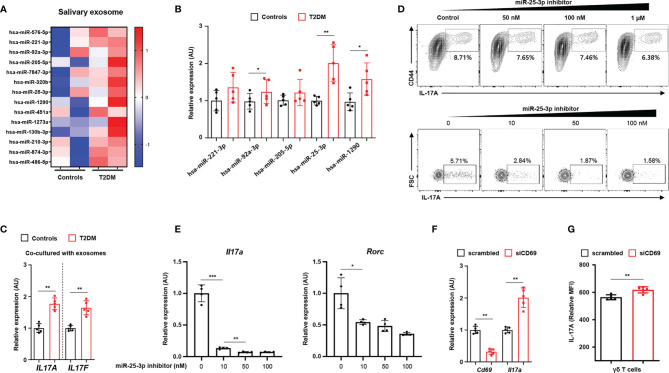
Salivary exosomal miRNA profiles in healthy individuals and in patients with type 2 diabetes. **(A)** Heatmap showing differentially enriched miRNAs in salivary exosomes from patients with type 2 diabetes as compared with those from normoglycemic healthy individuals. **(B)** Expression of differentially enriched miRNAs in salivary exosomes was measured by real-time PCR. **(C)** Co-culturing γδ T cells with salivary exosomes (from either healthy individuals or patients with type 2 diabetes) with anti-CD3 (2 μg/mL), anti-IL-2 (10 ng/mL), and isopentenyl pyrophosphate (5 μg/mL). **(D)** The population of IL‐17A-producing cells under culture conditions of γδ T cell activation at the indicated concentrations of miR-25-3p inhibitors. **(E)** Transcription of *Il17a* and *Rorc* in differentiated CD4^+^ T cells under T_H_17 differentiation-inducing culture conditions at the indicated concentrations of miR-25-3p inhibitors. **(F, G)** γδ T cells were transfected with either control siRNA or anti-CD69 siRNA for 48 hr. Expression levels of IL-17A were measured by using qPCR and FACS analysis. Data represent mean values of more than three independent experiments. Data are expressed as the mean ± SEM. *p < 0.05, **p < 0.01, ***p < 0.001 **(B, C, F, G)**, two‐tailed *t* tests; **(E)**, one‐way ANOVA.

We characterized exosomes from whole saliva (from healthy individuals and T2D patients) by transmission electron microscopy and by immunoblot analysis (for exosomal markers, including CD63 and TSG101). We found that exosome sizes were similar in both groups (52.86 ± 2.97 nm in healthy individuals *vs.* 52.51 ± 3.64 mm in T2D patients; P = 0.836). We also measured similar levels of CD63 and TSG101 in both groups and detected neither ER (GRP94) nor Golgi (GM130) markers in either group (**Supplementary Figure 3F**). By performing RNA sequencing, we identified 14 miRNAs that were more highly expressed in the salivary exosomes from T2D patients than in those from healthy individuals (**Figure 2A**; we excluded transcripts with 0–10 read counts to avoid possible artefacts) (16). We validated the results for five of these miRNAs by using quantitative real-time PCR to measure expression in salivary exosomes of 15 T2D patients and 15 healthy individuals. The real-time PCR results for three of the tested miRNAs (miR-92-3p, miR-25-3p, and miR-1290) agreed with the RNA sequencing results (**Figure 2B**). Given that miR-1290 expression is undetectable in mice (17), we investigated the roles of both miR-92-3p and miR-25-3p in γδ T cell production of IL-17; these miRNAs are implicated in inflammation and bone destruction of periodontitis. Of these two miRNAs, only miR-25-3p is predicted to function in lymphocytes, PBMCs, and tonsils (**Supplementary Figure 3A**). Treatment with salivary exosomes from T2D patients increased transcription of both *IL17A* and *IL17F* in γδ T cells (**Figure 2C**). Treatment with miR-25-3p inhibitors suppressed both IL-17 production in γδ T cells and differentiation of T_H_17 cells in a dose-dependent manner (**Figure 2D** and **Supplementary Figure 4B**). Moreover, expression levels of both *Il17a* and *Rorc* were significantly downregulated by miR-25-3p inhibitors during T_H_17 differentiation *in vitro* (**Figure 2E**). We next identified potential mRNA targets by using the TargetScan algorithm to search for miR-25-3p-binding sites in the 3′-UTR of mRNAs (**Supplementary Figure 3C**) (18). We focused on CD69 mRNA, which is predicted to bind miR-25-3p and to encode a protein that functions in both T cell differentiation and the inflammatory response (**Supplementary Figure 4D**) (19, 20). To confirm whether CD69 is involved in IL-17 production in γδ T cells, we inhibited CD69 expression by transfecting γδ T cells with CD69 mRNA-targeting small interfering RNAs and then inducing activation *in vitro*. Knockdown of CD69 increased IL-17 production in γδ T cells significantly (**Figures 2F, G** and **Supplementary Figure 4E**). We measured cell viability in response to the miR-25-3p inhibitor using an MTT assay, and we found no significant effect of miR-25-3p on γδ T cell viability (**Supplementary Figure 4F**). Collectively, these results indicate that exosomal miR-25-3p is highly enriched in saliva of obese T2D patients and that down-regulation of CD69 (a target of miR-25-3p) increases IL-17 production in γδ T cells.

### Inhibition of miR-25-3p Attenuates Local Inflammation in Mice With Periodontitis

**Figure 3:**
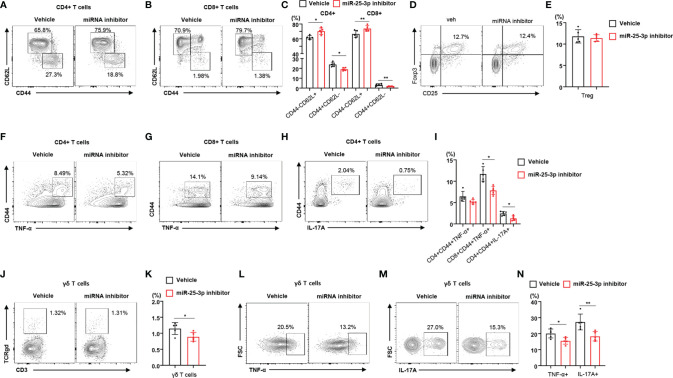
Inhibition of miR-25-3p attenuates periodontal inflammation in male mice with diet-induced obesity and periodontitis. **(A–C)** Representative flow cytometry plots and percentages of CD44^+^CD62L^−^ and CD44^−^CD62L^−^ cells in the CD4^+^ and CD8^+^ T cell populations from submandibular lymph nodes of either vehicle-treated (n = 5) or miR-25-3p inhibitor-treated (n = 5) male mice with periodontitis 9 days after ligature placement. **(D, E)** Representative flow cytometry plots and frequencies of CD4^+^CD25^+^Foxp3^+^ regulatory T cells in HFD-fed mice treated with either miR-25-3p inhibitor or vehicle. **(F–I)** Percentages of TNF-α- or IL-17A-producing cells in the population of CD4^+^ or CD8^+^ T cells in periodontitis-induced mice treated with either miR-25-3p inhibitor or vehicle. **(J, K)** Percentages of γδ T cells in submandibular lymph nodes of periodontitis-induced mice treated with either miR-25-3p inhibitor or vehicle. **(L–N)** Representative flow cytometry plots and percentages of TNF-α- or IL-17A-producing cells in the population of γδ T cells from periodontitis-induced mice treated with either miR-25-3p inhibitor or vehicle. Data represent mean values of more than three independent experiments. Data are expressed as the mean ± SEM. *p < 0.05, **p < 0.01 **(C, E, I, K, N)**, two‐tailed *t* tests.

To determine the effect of miR-25-3p inhibition on local inflammation in mice with periodontitis, we subcutaneously injected miR-25-3p inhibitors into the right side of the lower jaw three times per week for 2 weeks. We found that the body weight of mice treated with miR-25-3p inhibitors was not significantly different from that of untreated mice (**Supplementary Figure 5A**). Since the miR-25-3p inhibitors attenuated T_H_17 differentiation *in vitro*, we investigated the immunophenotypes of lymphocytes in control (non-ligated) and regional lymph nodes of HFD-fed mice treated with either miR-25-3p inhibitors or vehicle. Treatment with miR-25-3p inhibitors did not change the populations of TNF-α- or IL-17A-producing CD4^+^ and CD8^+^ T cells in the control (non-ligated) lymph nodes of diet-induced obese mice (**Supplementary Figure 5B**). However, we found a lower percentage of memory T cells in the regional lymph nodes of HFD-fed periodontal mice treated with miR-25-3p inhibitors (compared to those treated with vehicle), and a higher percentage of naïve T cells (**Figures 3A–C**). However, the percentage of regulatory T cells in mice treated with miR-25-3p inhibitors was the same as that in mice treated with vehicle (**Figures 3D, E**). Using intracellular cytokine staining, we found that the percentages of TNF-α- or IL-17A-producing memory CD4^+^ and CD8^+^ T cells were significantly lower in the regional lymph nodes of mice treated with miR-25-3p inhibitors (**Figures 3F–I**). Furthermore, the percentage of γδ T cells was lower in the regional lymph nodes of periodontal mice treated with miR-25-3p inhibitors (**Figures 3J, K**). Additionally, the percentage of TNF-α- or IL-17-producing γδ T cells was significantly lower in the regional lymph nodes of mice treated with miR-25-3p inhibitors than in mice treated with vehicle (**Figures 3L–N**).

### Inhibition of miR-25-3p Alleviates Ligature-Induced Periodontal Bone Loss in Mice With Diet-Induced Obesity

**Figure 4:**
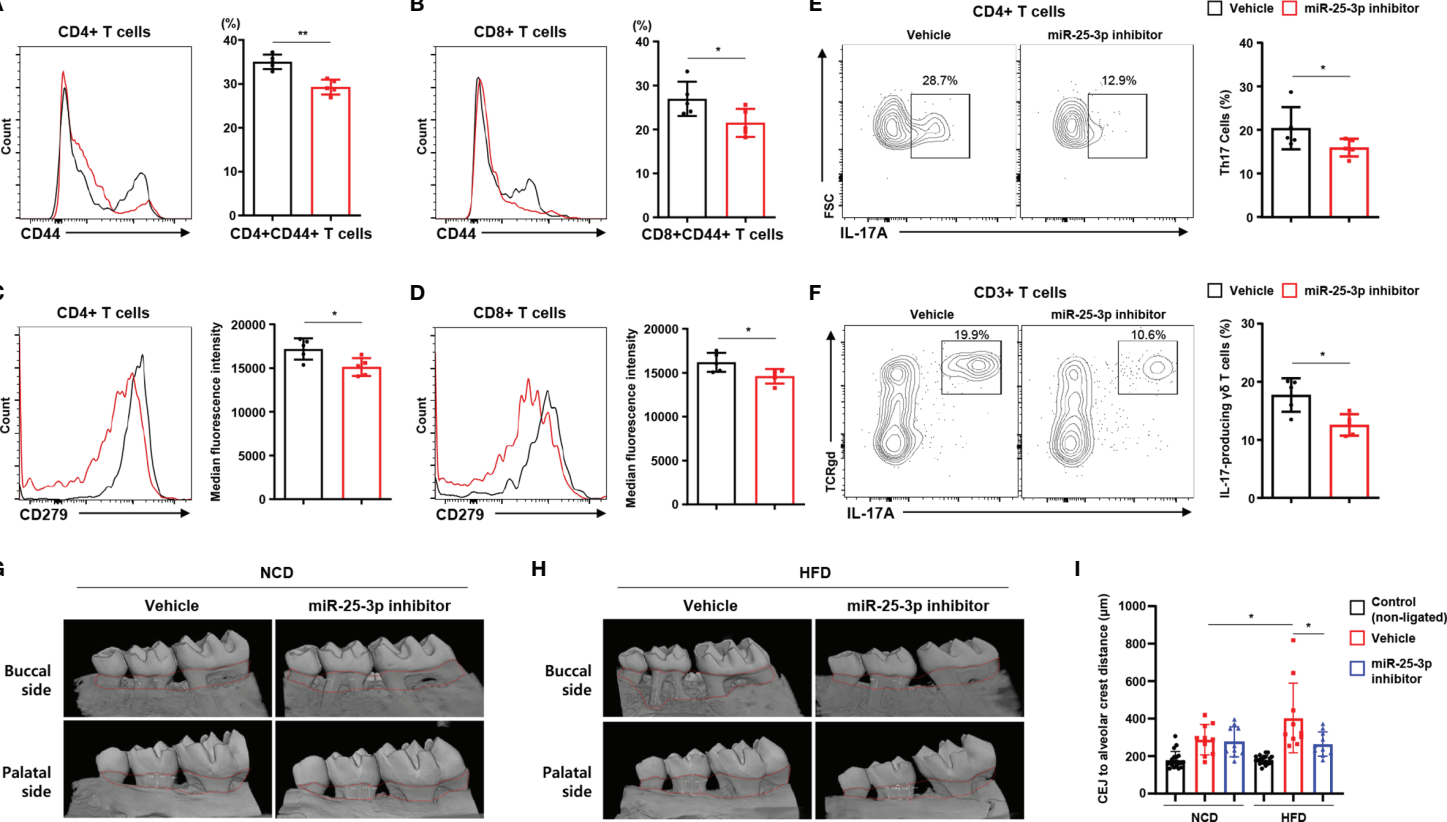
Treatment with miR-25-3p inhibitor decreases the populations of effector T cells and IL-17-producing cells in inflamed gingiva of obese male mice with periodontitis. **(A, B)** Size of the CD44^+^ population in inflamed gingiva-infiltrating CD4^+^ and CD8^+^ T cells from periodontitis-induced obese male mice treated with either miR-25-3p inhibitor (n = 5) or vehicle (n = 5). **(C, D)** Median fluorescence intensity of CD279 was measured in inflamed gingiva-infiltrating CD4^+^ and CD8^+^ T cells of periodontitis-induced obese mice treated with either miR-25-3p inhibitor or vehicle. **(E)** Representative flow cytometry plots and percentages in inflamed gingiva-infiltrating IL-17A-producing cells of the population of CD4^+^ T cells in periodontitis-induced obese mice treated with either miR-25-3p inhibitor or vehicle. **(F)** Representative flow cytometry plots and percentages in inflamed gingiva-infiltrating IL-17A-producing cells of the population of γδ T cells from periodontitis-induced obese mice treated with either miR-25-3p inhibitor or vehicle. **(G, H)** Synchrotron radiation micro-computed tomography analysis of periodontitis-induced bone loss in mice fed either a NCD or HFD and treated with either miR-25-3p inhibitor or vehicle. The upper red-dotted line indicates the cemento-enamel junction; the lower red-dotted line indicates the alveolar bone crest (left panel). **(I)** The distances from cemento-enamel junctions to alveolar bone crests were measured around the second molars on transaxial and sagittal sections along the buccal, palatal, and interdental axes. Distances were statistically analyzed in mice fed either a NCD or a HFD and treated with either miR-25-3p inhibitor or vehicle. Data represent mean value of more than three independent experiments. Data are expressed as the mean ± SEM. *p < 0.05, **p < 0.01 **(A–F)**, two‐tailed *t* tests; **(G–I)**, one‐way ANOVA.

Next, we investigated lymphocytes in inflamed gingiva of HFD-fed mice treated with either miR-25-3p inhibitors or vehicle. Of the population of CD4^+^ and CD8^+^ T cells that had infiltrated the inflamed gingiva, the percentage of CD44^+^ memory T cells was lower in periodontitis-induced obese mice treated with miR-25-3p inhibitor (**Figures 4A, B**). CD279 (a marker of T cell activation and exhaustion) was downregulated in gingiva-infiltrating CD4^+^ and CD8^+^ T cells of obese mice treated with miR-25-3p inhibitor (**Figures 4C, D**). The percentages of gingival T_H_17 cells and IL-17-producing γδ T cells were significantly lower in periodontitis-induced obese mice treated with miR-25-3p inhibitor (**Figures 4E, F**). These data suggest that inhibition of miR-25-3p ameliorates the local inflammatory response in periodontal mice with both diet-induced obesity and insulin resistance. Since miR-25-3p mediated IL-17-associated inflammation in periodontitis, we investigated the role of miR-25-3p on periodontal alveolar bone loss. We used synchrotron radiation micro-computed tomography (SR-µCT) to measure changes in distances between cemento-enamel junctions and alveolar bone crests in NCD-fed and HFD-fed periodontal mice treated with either miR-25-3p inhibitors or vehicle. Mice fed a HFD were more susceptible to ligature-induced periodontal bone loss than mice fed a NCD (**Figures 4G–I**). Local injection of miR-25-3p inhibitors significantly attenuated ligature-induced periodontal alveolar bone loss in HFD-fed mice, but not in NCD-fed mice (**Figures 4G–I**). Taken together, these results suggest that suppression of local T_H_17 inflammation by miR-25-3p inhibitors alleviates ligature-induced periodontal bone loss in mice with diet-induced obesity.

## Discussion

**Figure 5:**
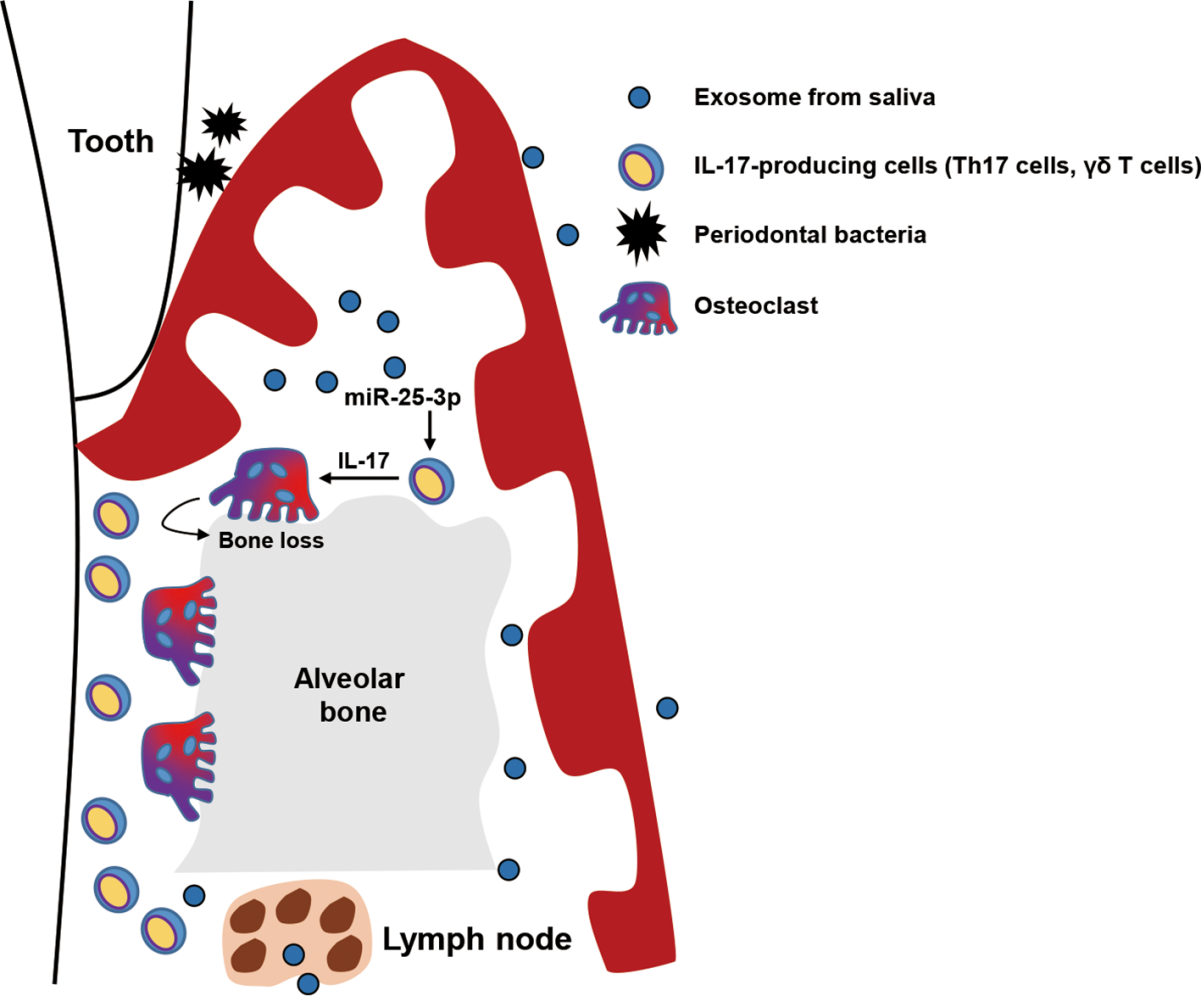
Graphical abstract. Exosomal miR-25-3p in saliva is highly enriched in patients with type 2 diabetes. Salivary exosomal miR-25-3p promotes local inflammation and periodontal bone loss in mice by activating T_H_17 cells and γδ T cells.

In this study, we investigated the role of salivary exosomal miRNAs in progression of periodontitis in mice with diet-induced obesity and insulin resistance. We found that miR-25-3p is more enriched in salivary exosomes of T2D patients than in those of healthy individuals. We also found that salivary exosomal miR-25-3p downregulates CD69 in IL-17-producing γδ T cells, which is implicated in periodontal inflammation and bone loss in obese mice with ligature-induced periodontitis (**Figure 5**). Moreover, local treatment with miR-25-3p inhibitors attenuated IL-17-mediated periodontal inflammation and alveolar bone loss in mice with periodontitis and diet-induced obesity.

Saliva is a highly complex mixture (which includes exosomes, immunoglobulins, enzymes, hormones, nucleic acids, and bacteria) that reflects physiological and pathological states in humans (21). Salivary components are biomarkers of many periodontal diseases (22, 23). Exosomes (a salivary component) function to secrete RNAs, proteins, enzymes, and lipids to cells at either proximal or distal locations (24), but the contribution of salivary exosomes for the development of periodontal diseases has not been determined. In this study, we showed that salivary exosomal miR-25-3p exacerbates local inflammation and bone loss by modulating γδ T cell activation in mice with diet-induced obesity and periodontitis. Thus, obesity-induced insulin resistance changes the exosomal miRNA profile of saliva, which may contribute to progression of periodontitis. Therefore, we suggest that exosomal miR-25-3p in saliva is a therapeutic target and a useful biomarker for diabetes-associated periodontitis.

The miR-25 family is highly conserved in vertebrates and is predicted to bind the same mRNA targets as other miRNA members of this seed family (TargetScanHuman version 7.1). MiR-25-3p has context-dependent functions in many kinds of diseases (including cancer, inflammation, and metabolic disease). Exosome-derived miR-25-3p stimulates secretion of pro-inflammatory cytokines from tumor-associated macrophages, resulting in liposarcoma progression (25). MiR-25-3p promotes proliferation of triple-negative breast cancer by directly targeting B-cell translocation gene 2 (26) and promotes osteoclast differentiation by regulating the expression of nuclear factor I X (27). MiR-25 also directly reduces insulin expression, whereas miR-25 inhibition (by using corresponding antagomiRs) promotes insulin expression in the INS-1 cell line (28). Moreover, miR-25 is associated with residual beta-cell function and poor glycemic control during disease progression in children with new-onset type 1 diabetes (29). On the other hand, miR-25-3p attenuates oxidized low-density lipoprotein-mediated coronary vascular endothelial cell inflammation by targeting Adam10 in *ApoE^–/–^* mouse models of atherosclerosis (30). However, the role of miR-25-3p in progression of periodontitis remains to be established. We revealed that miR-25-3p inhibitors suppress activation of γδ T cells *in vitro* and attenuate progression of periodontal inflammation and alveolar bone loss by reducing IL-17-producing T cells in a mouse model of ligature-induced periodontitis.

A main strength of this translational study is the discovery that salivary exosomal miR-25-3p in insulin resistance-associated periodontitis in obese mice leads to periodontal inflammation and bone loss by increasing the population of IL-17-producing cells. This work may facilitate application of new therapeutics for periodontal disease. However, this study also has several limitations. First and most importantly, the delivery mechanism of salivary exosomes to periodontal immune cells is unclear. Given that exosomal cargo (including miRNAs) can function after delivery, the biogenesis and transfer mechanism of salivary exosomes in the development of periodontitis must be defined. Second, the differences between T2D-patient salivary exosome profiles and healthy-individual exosome profiles may be a consequence, not a cause, of periodontal inflammation progression. Third, this study did not consider the role of obesity-induced adipokines on periodontal inflammation. Systemic inflammatory cytokines induced by diet-induced obesity and insulin resistance can be confounding factors in local inflammation, which may be regulated by exosomal miR-25-3p during progression of periodontitis. Finally, we could not evaluate the populations and activities of periodontal immune cells in human patients, so further investigation is required to translate these preclinical results from mouse models to humans.

In conclusion, exosomal miR-25-3p is enriched in the saliva of T2D patients. Our study also provides critical insights into the regulation of IL-17–mediated local inflammation by exosomal miR-25-3p during the development of periodontitis. We revealed that inhibition of miR-25-3p attenuates the progression of periodontal inflammation and bone loss by deactivating IL-17-producing γδ T cells. However, additional mechanistic studies are needed to confirm whether administration of miR-25-3p inhibitors effectively treats diabetes-associated periodontitis in humans.

## Data Availability Statement

The datasets presented in this study can be found in online repositories. The names of the repository and accession number can be found below: https://www.ncbi.nlm.nih.gov/geo/query/acc.cgi?acc=GSE189107.

## Ethics Statement

The studies involving human participants were reviewed and approved by Chungnam National University Hospital. The patients/participants provided their written informed consent to participate in this study. The animal study was reviewed and approved by Chungnam National University School of Medicine

## Author Contributions

JT and JM maintained the mouse colony, induced and managed diet-induced obese mice, performed flow cytometry experiments, and administered miR-25-3p inhibitors to mice. J-SB and JM purified exosomes from human saliva and performed the RNA-seq analysis of human salivary exosomes. S-HL observed salivary exosomes using transmission electron microscopy. JC generated the miR-25-3p inhibitor. Y-GK conducted SR-µCT analysis of alveolar bone. J-SB, Y-GK, and S-HL designed the study, analyzed the data, and wrote the article. JT and HL performed *in vitro* experiments using γδ T cells. J-SB and H-SY performed the bioinformatic analysis. Y-GK and H-SY are the guarantors of this work and, as such, had full access to all the data in the study and take responsibility for the integrity of the data and the accuracy of the data analysis. All authors contributed to the article and approved the submitted version.

## Funding

This work was supported by the Bumsuk Academic Scholarship Foundation. H-SY was supported by the Basic Science Research Program, through the National Research Foundation of Korea, and funded by the Ministry of Science, ICT, and Future Planning, Korea (NRF‐2021R1A5A8029876 and NRF‐2019M3E5D1A02068575) and the CNUH Research Fund, 2021. Y-GK was supported by a National Research Foundation of Korea grant, funded by the Korea government (NRF‐2020R1A2C1013306). J-SB was supported by the National Research Foundation of Korea grant funded by the Korea government (NRF-2020R1C1C1006757). HL and JT were supported by BK21 FOUR Program by Chungnam National University Research Grant, 2021

## Conflict of Interest

Author JC is employed by Panagene Inc.

The remaining authors declare that the research was conducted in the absence of any commercial or financial relationships that could be construed as a potential conflict of interest.

## Publisher’s Note

All claims expressed in this article are solely those of the authors and do not necessarily represent those of their affiliated organizations, or those of the publisher, the editors and the reviewers. Any product that may be evaluated in this article, or claim that may be made by its manufacturer, is not guaranteed or endorsed by the publisher.
