# Supplementary material for: Effect of Salivary Exosomal miR-25-3p on Periodontitis With Insulin Resistance
Source: Front Immunol. 2022 Jan 7;12:775046. doi: 10.3389/fimmu.2021.775046 (PMC8777127; doi:10.3389/fimmu.2021.775046)
Supplement: Supplementary file 1 [file DataSheet_1.pdf]

Supl Fig. 1

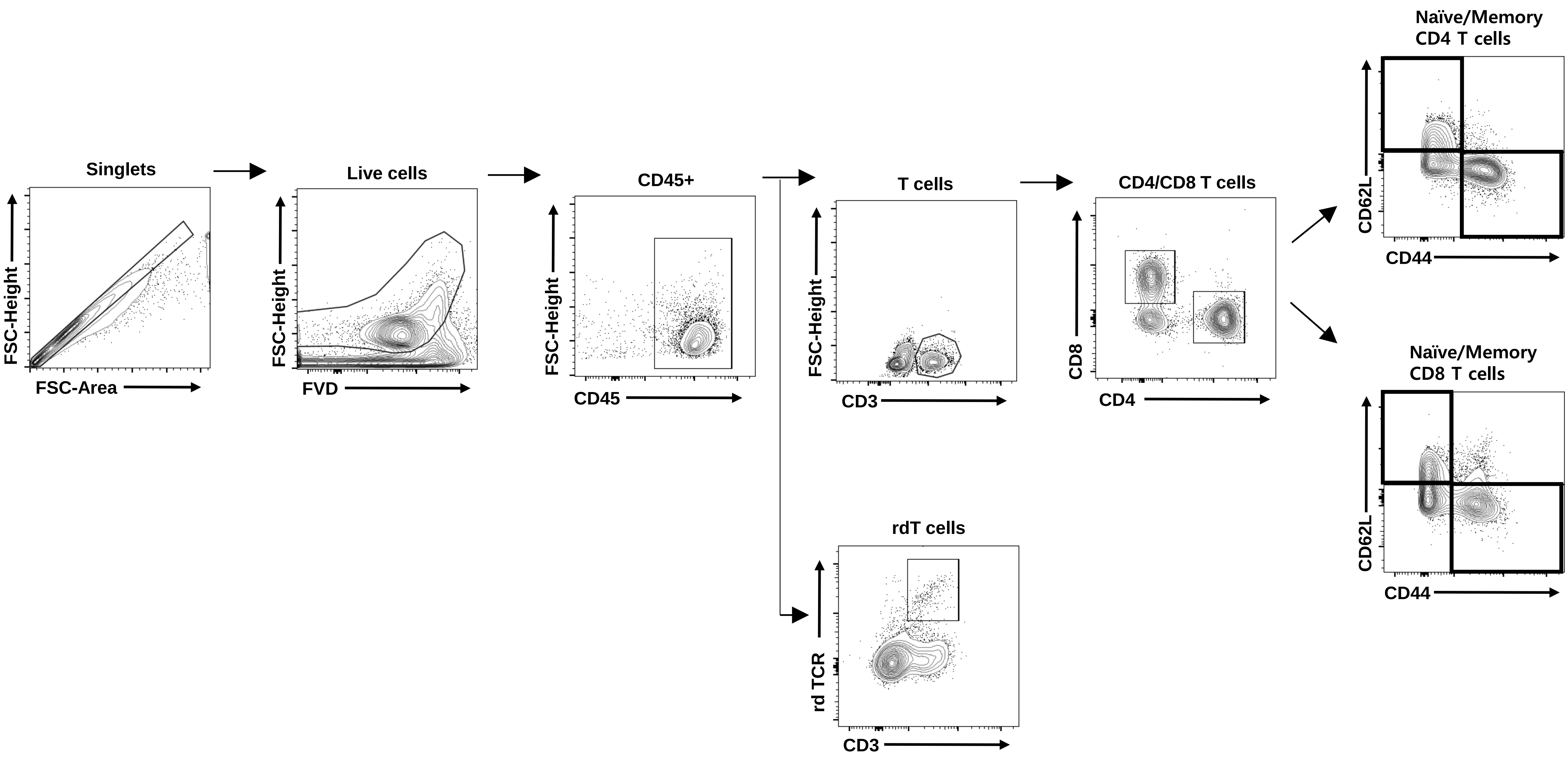

Singlets
FSC-Height
FSC-Area
Live cells
FSC-Height
FVD
CD45+
FSC-Height
CD45
T cells
FSC-Height
CD3
CD4/CD8 T cells
CD8
CD4
Naïve/Memory
CD4 T cells
CD62L
CD44
Naïve/Memory
CD8 T cells
CD62L
CD44
rdT cells
rd TCR
CD3

Supl Fig. 2

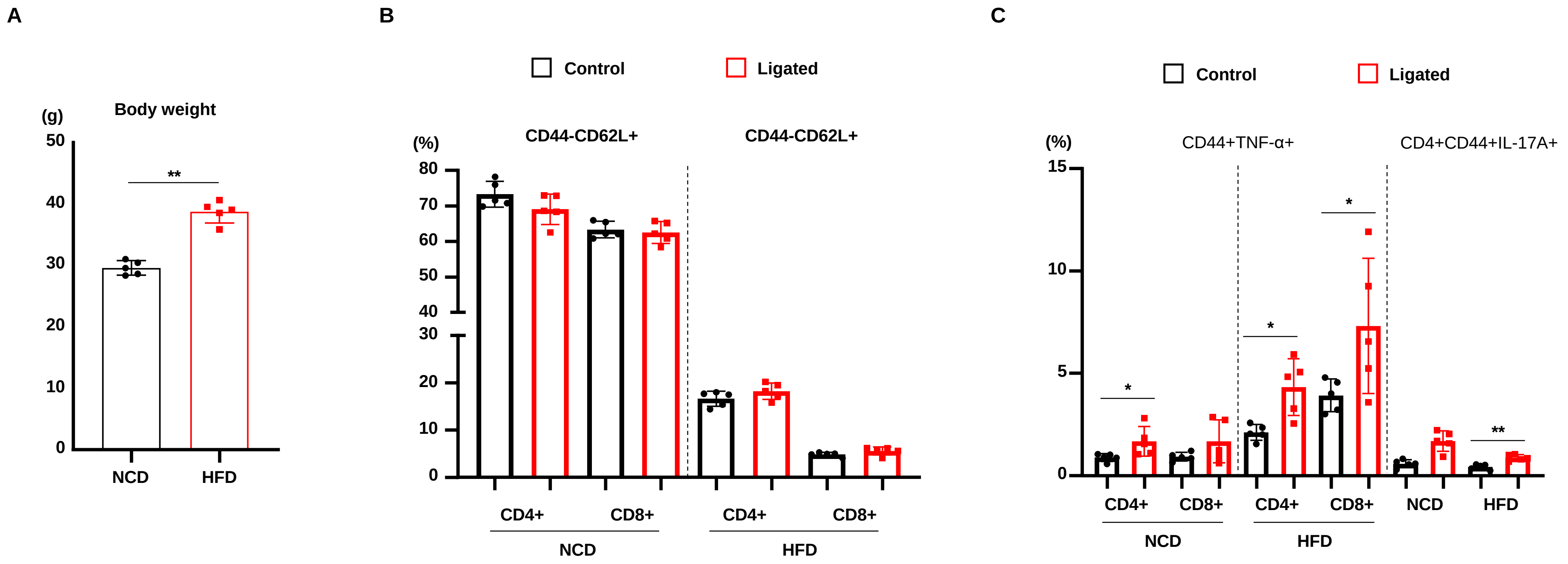
A
Body weight
(g)
50
40
30
20
10
0
**
NCD
HFD
B
Control
Ligated
(%)
CD44-CD62L+
CD44-CD62L+
80
70
60
50
40
30
20
10
0
CD4+
CD8+
CD4+
CD8+
NCD
HFD
C
Control
Ligated
(%)
CD44+TNF-α+
CD4+CD44+IL-17A+
15
10
5
0
*
*
*
**
CD4+
CD8+
CD4+
CD8+
NCD
HFD
NCD
HFD

Supl Fig. 3

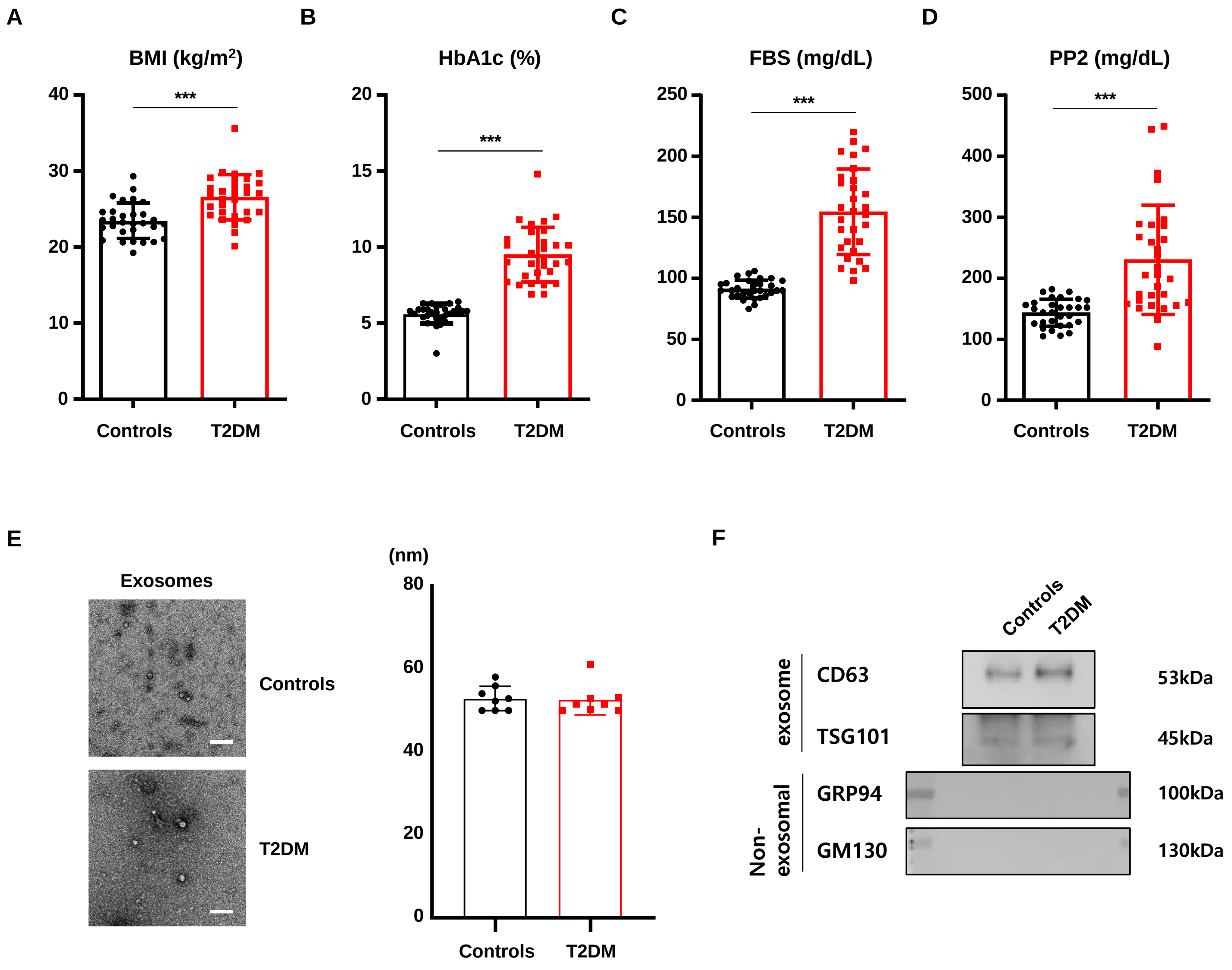
A
BMI (kg/m²)
***
Controls
T2DM
B
HbA1c (%)
***
Controls
T2DM
C
FBS (mg/dL)
***
Controls
T2DM
D
PP2 (mg/dL)
***
Controls
T2DM
E
Exosomes
Controls
T2DM
(nm)
Controls
T2DM
F
Controls
T2DM
exosome
CD63
TSG101
Non-exosomal
GRP94
GM130
53kDa
45kDa
100kDa
130kDa

Supl Fig. 4

A

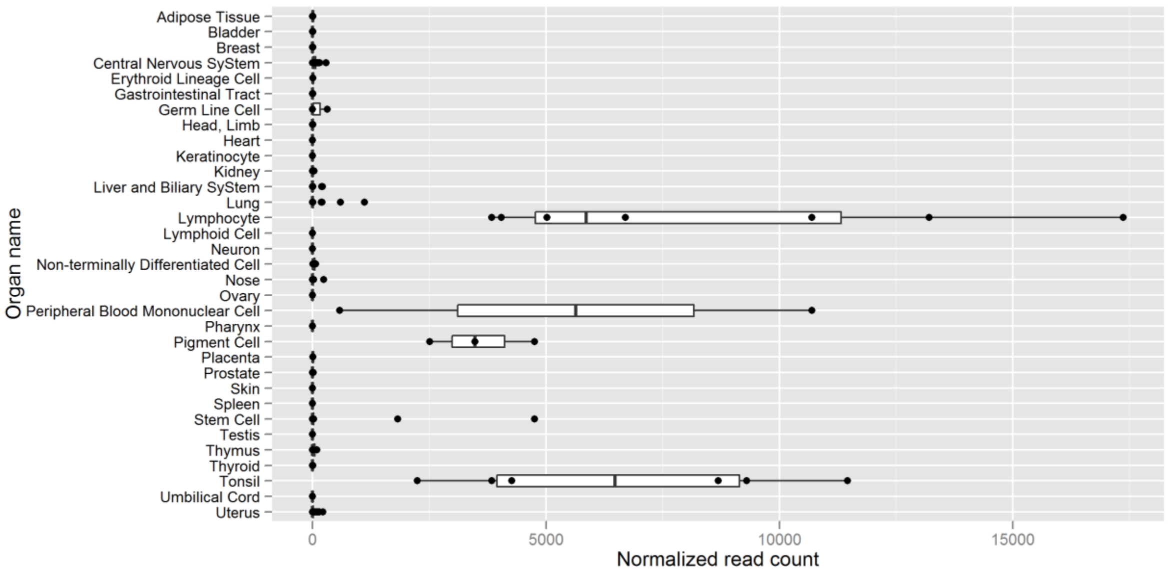

B

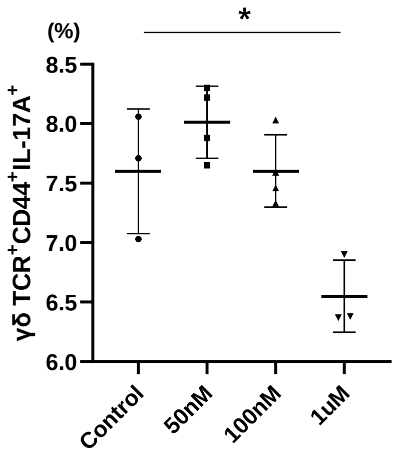

D

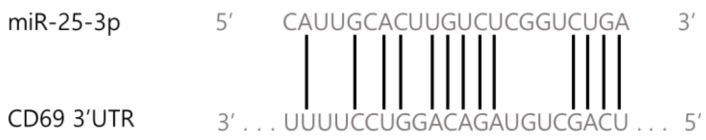

C

Target Rank	Target Score	miRNA Name	Gene Symbol	Gene Description
1	100	hsa-miR-25-3p	CD69	CD69 molecule
2	100	hsa-miR-25-3p	SLC12A5	solute carrier family 12 member 5
3	100	hsa-miR-25-3p	MAN2A1	mannosidase alpha class 2A member 1
4	100	hsa-miR-25-3p	G3BP2	G3BP stress granule assembly factor 2
5	100	hsa-miR-25-3p	FBXW7	F-box and WD repeat domain containing 7
6	99	hsa-miR-25-3p	PIKFYVE	phosphoinositide kinase, FYVE-type zinc finger containing
7	99	hsa-miR-25-3p	MAP2K4	mitogen-activated protein kinase kinase 4
8	99	hsa-miR-25-3p	B3GALT2	beta-1,3-galactosyltransferase 2
9	99	hsa-miR-25-3p	FNIP1	folliculin interacting protein 1
10	99	hsa-miR-25-3p	SLC17A6	solute carrier family 17 member 6

E

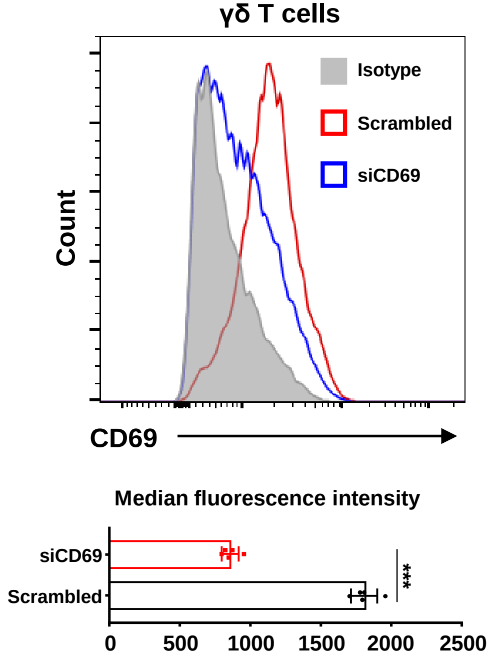

F

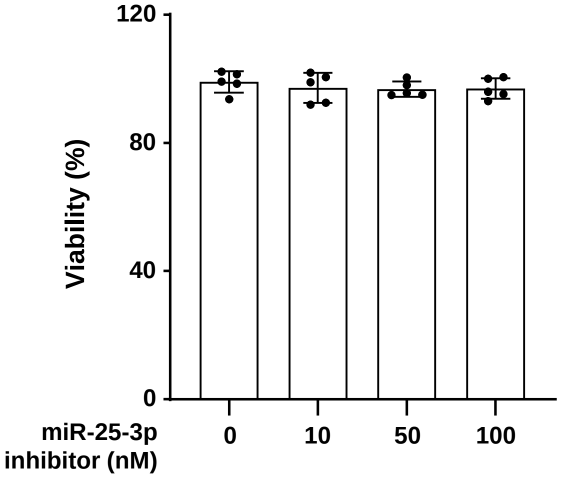

Supl Fig. 5

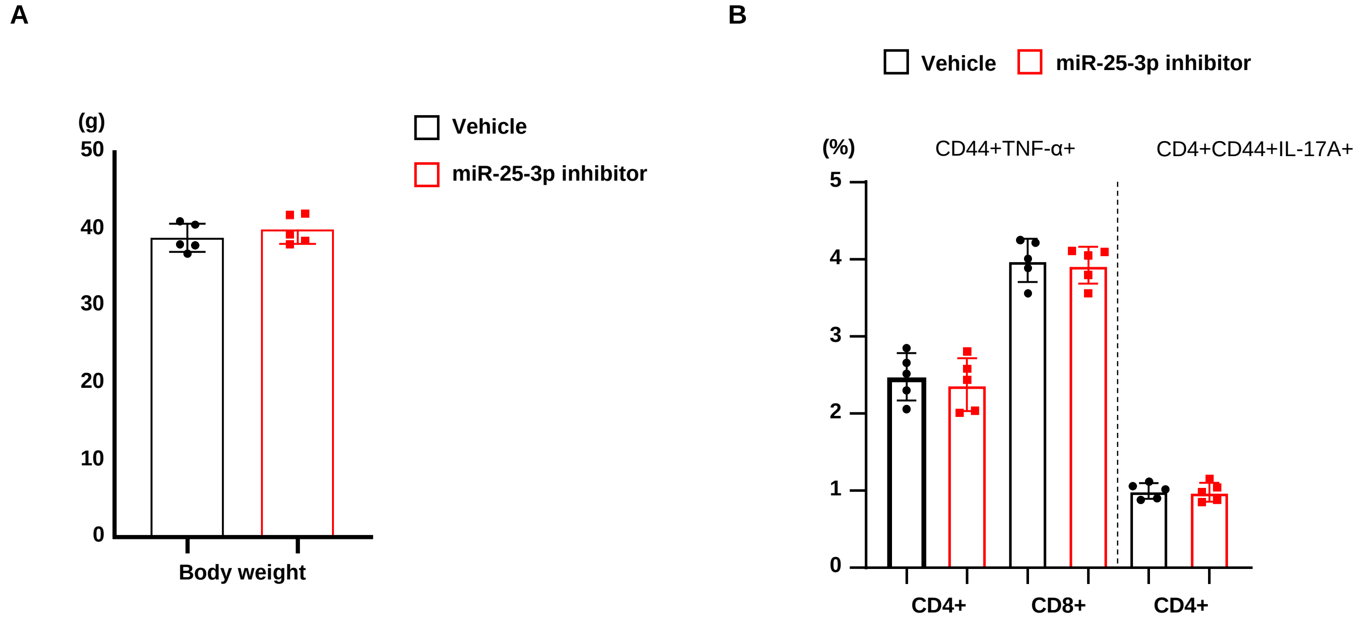

A
(g)
50
40
30
20
10
0
Body weight
Vehicle
miR-25-3p inhibitor
B
Vehicle
miR-25-3p inhibitor
(%)
CD44+TNF-α+
CD4+CD44+IL-17A+
5
4
3
2
1
0
CD4+
CD8+
CD4+
